# Handgrip strength measurement protocols in individuals with Down syndrome: a systematic review and meta-regression: Handgrip strength measurement protocols and Down syndrome

**DOI:** 10.1080/07853890.2025.2453077

**Published:** 2025-01-22

**Authors:** Geiziane L. R. Melo, Rafaela C. Espírito Santo, Viney P. Dubey, Cesar Agostinis-Sobrinho

**Affiliations:** Health Research and Innovatioin Science Centre, Klaipeda University, Klaipeda, Lithuania

**Keywords:** Intellectual disability, muscle strength dynamometer, risk assessment, trisomy 21, muscle strength

## Abstract

**Background:**

Handgrip strength (HGS) serves as a robust predictor of overall strength across various populations, including individuals with Down Syndrome (DS).

**Objective:**

To analyze the HGS measurement protocols used in studies involving individuals with DS.

**Methods:**

Primary sources were sourced from six databases: PubMed, Scopus, Ovid, Embase, ERIC, and Web of Science, spanning from inception to 23rd December 2023. Inclusion criteria focused on individuals with DS, compared with control groups, and examined HGS measurement protocols and outcomes. Meta-regression was utilized to assess bias associated with HGS values concerning different measurement protocols.

**Results:**

Out of 29 studies involving 1816 participants, most controlled for body position (65%), arm position (82%), elbow position (82%), wrist position (62%), handgrip duration (55%), hand adjustment to dynamometer (62%), verbal encouragement (75%), and familiarization (44.8%). The number of reported variables in the HGS protocol was significantly associated with an increase in HGS, with a mean estimate of 20.59 units (SE = 2.59, *p* < 0.0001, 95% CI [15.49–25.68]), though there was notable heterogeneity (*I*^2^ = 94.33%). The spline regression analysis showed that the model explained 82.66% of the variation in HGS, with adults having 47.61 units higher HGS than children (*p* = 0.0009), while obesity was linked to a decrease of 15.68 units (*p* = 0.0675). Sample size and group had no significant effects.

**Conclusion:**

Overall, protocols for assessing HGS in DS studies are comprehensive yet heterogeneous. Higher HGS values correlated with adherence to standard protocols.

## KEY MESSAGES

- Handgrip strength values were higher when studies adhered to the standard protocol for measuring handgrip strength.
- Standardizing handgrip strength measurement protocols and establishing controlled methods are crucial for improving the accuracy of assessing handgrip strength in individuals with DS.
- Age and BMI can also contribute to different handgrip estimates using independent measurement protocols.
- Visual feedback, verbal encouragement and familiarization are essential for understanding handgrip strength measurement protocol variables in individuals with DS.

## Introduction

Down syndrome (DS) is a chromosomal condition characterized by a third copy of chromosome 21 and is a genetic cause of intellectual disability, affecting approximately 1 in 700 newborns in the United States [1,2]. Since 1950, there has been an exponential increase in the life expectancy of individuals with DS due to improvements in health and social care [3]. This highlights the ongoing importance of delivering inclusive and equitable healthcare, ensuring that individuals with DS continue to have access to the support and resources they need [1,2].

Notably, people with DS not only experience challenges related to muscle mass, strength maintenance, and physical performance but also face an elevated risk of developing metabolic syndrome, obesity, diabetes mellitus, and an increased risk of sarcopenia compared to the general population [4–6]. High levels of obesity and low levels of physical activity are key issues in the lives of individuals with DS [7].

Previous studies have used handgrip (HGS) as a strong predictor of mortality risk in the general population [7,8]. Unfortunately, there are currently no studies that focus exclusively on individuals with DS. However, previous studies have used HGS to assess physical decline in adults with intellectual disabilities (ID), including DS, highlighting that reduced muscle strength is associated with increased frailty and higher mortality risk [9,10]. Utilizing HGS as a screening tool can contribute significantly to early detection and intervention strategies aimed at preserving overall health and quality of life in individuals with DS [5,11]. It is a tool for assessing overall strength and a reliable indicator of physical well-being in individuals with DS [12,13].

In contrast, individuals with DS exhibit diminished HGS as compared with the general population, regardless of age [4,11]. Previous research has shown the critical role of HGS in assessing overall muscle strength, serving as an indicator of accelerated aging, onset of dementia, and a higher risk of future functional disability within this population [5,11,14]. It is simple, rapid, and cost-effective, making HGS an ideal metric for application in clinical and large-scale research settings [8,15,16]. Notably, HGS exhibits high levels of validity and reliability in individuals with DS [12,13].

However, in research settings, the measurement protocol for characterizing HGS may vary significantly, encompassing differing elbow, arm, wrist, and lower extremity positions, the number of tests conducted, and the stimulus during the evaluation [17–19]. Familiarization, verbal encouragement, and visual feedback are fundamental tools for understanding and performing maximum HGS [20,21]. Therefore, it is imperative to employ straightforward language and provide visual demonstrations to explain the requisite movements to ensure comprehension in individuals with DS [22,23]. Nevertheless, studies frequently fail to provide adequate information regarding the available protocols [17,18].

Given this context, the measurement heterogeneity protocol may impact the estimates in studies investigating associations between HGS measurement protocols and lower HGS outcomes in individuals with DS. Consequently, it is necessary to analyze the available evidence to identify the heterogeneity in the protocols used in such studies. The aim of this systematic review was to analyze the HGS measurement protocols used in studies involving individuals with DS. We also included assessing the potential bias associated with HGS values resulting from the use of various measurement protocols.

## Methods

This systematic review was conducted according to the Preferred Reporting Items for Systematic Reviews and Meta-Analyses (PRISMA) guidelines [24] and was previously registered on PROSPERO (registration number CRD42023485653).

### Search strategy

**Figure 1.**
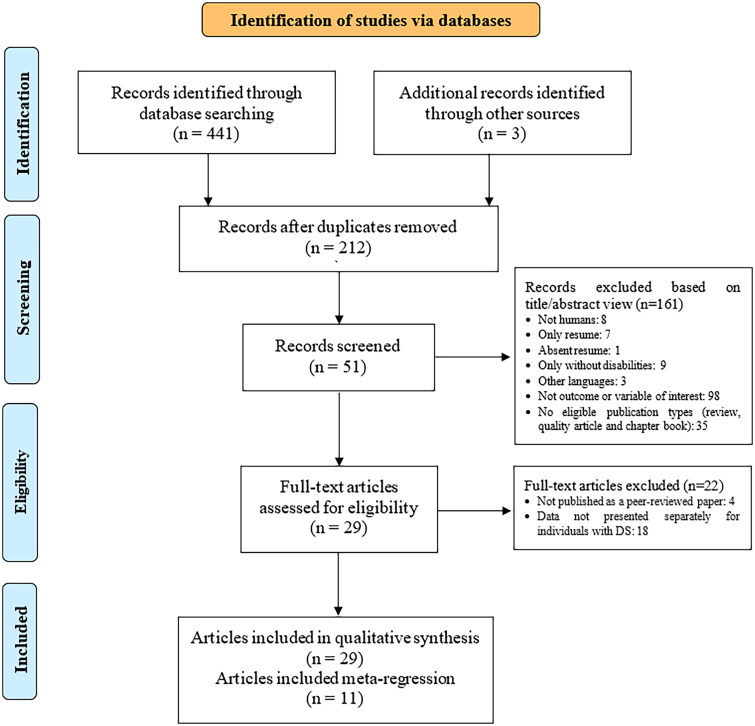
PRISMA flowchart for study selection.

The search spanned PubMed, Scopus, Ovid, Embase, ERIC, and Web of Science databases up to the 25th of October and 31st December 2023, without restrictions on publication year (see Figure 1). The keywords were initially chosen from MeSH terms and subsequently adjusted to guarantee the inclusion of all relevant studies. Electronic databases were searched using various combinations of the following sets of keywords: (1) ‘Down syndrome’ OR ‘trisomy 21’; AND (2) ‘hand’ OR ‘handgrip’ OR ‘grip’ OR ‘grasp’ OR ‘dynamometer’ OR ‘hand grip’ AND (3) ‘force’ OR ‘strength’ OR ‘isometric’ OR ‘strength test’ OR ‘strength measure’ OR ‘muscular test’. The ‘AND’ operator was applied between the three keyword groups, while the ‘OR’ operator was used within each keyword group (Supplementary Table 1).

#### Eligibility criteria

Inclusion criteria were as follows: (1) Population: individuals with DS, regardless of age; (2) Intervention: not applicable; (3) Comparator: studies with or without a comparator group were included; (4) Outcomes: studies reporting HGS measurement protocols and outcomes; (5) Study designs: cross-sectional and longitudinal design; (6) Peer-reviewed papers published in English, Spanish, or Portuguese languages. All references were deduplicated in Endnote (Clarivate Analytics, New York, USA). Then, references were title and abstract screened independently by two reviewers; after all references were screened, any disputes were resolved through discussion. Thereafter, the same procedure was used for full-text screening. For the PRISMA checklist, see the supplementary Table 6.

### Data extraction

Data from studies were independently extracted by researchers GRM and R.E.S. The extracted measures included study design, participant characteristics (such as sample size, body mass index (BMI) classified according to the Centers for Disease Control and Prevention, age range or mean values, and standard deviation (SD) for age/sex distribution), type of dynamometer, hand laterality tested, body position, arm position, elbow position, wrist position, hand adjustment to the dynamometer, number of repetitions per arm, estimation of maximum HGS, duration of the HGS test, recovery time, and any other additional measures that could describe the protocol used in accordance with the Southampton protocol [17] and the American Society of Hand Therapists (ASHT) protocol [19]. HGS outcomes reported included dominant and non-dominant HGS, measured in kilograms of force, kilograms, or kPa (Table 1).

**Table 1.** Details and HGS protocols of the studies in individuals with and without Down syndrome.

Author	Study details	Size	Age	Sex	BMI	Dynamometer	HGS protocols	Handgrip outcomes	Newcastle_ Ottawa Scale
**Children (age ≤ 12)**									
Beqaj, 2018 [25]	Cross-sectional	44	Female: 12.47 ± 5.08 Male: 11.72 ± 5.08	Female:19 Male: 25	Female: 23.07 ± 6.51 Male: 19.08 ± 6.61	Jamar	Dominant hand. Three trials. Seated position. Shoulder adducted and neutrally rotated. Elbow in a 90° flexion. Neutral wrist position. Hand-adjustable in second position. Verbal encouragement. 3–5 s maintenance. 1 min. recovery. Higher value.	Female: 11.75 ± 5.22 Male: 11.50 ± 6.90	High
Morris, 1982 [26]	Cross-sectional	58	DS: 10.42 non-DS: 11	Experimental: Female: 15 Male: 13 Control: Female: 18 Male: 15	DS: 43.7 non-DS: 38.1	Lafayette.	Dominant hand. Three trials. Adjusted to hand size. Verbal encouragement. Higher value. Familiarization.	DS: 22.1 non-DS: 10.7	Medium
Priosti, 2013 [27]	Cross-sectional	56	DS: 7.75 ± 0.76 non-DS: 8.00 ± 0.84	DS: Female: 12 Male: 14 non-DS: Female: 15 Male: 15	–	Jamar.	Dominant hand. Three trials. Seated position. Shoulder adducted and neutrally rotated. Elbow in a 90° flexion. Wrist neutral. Hand-adjustable in second position. Verbal encouragement. 1 min recovery. Higher value.	DS: 9.5 non-DS: 15.9	Medium
Siyah, 2023 [28]	Cross-sectional	28	DS_CHD: 2.54 ± 1.10 DS_non-CHD: 2.48 ± 1.17	CHD: Female: 10 Male: 4 non-CHD: Female: 8 Male: 6	DS_CHD: 17.1 DS_non-CHD: 17.5	Fabrication Enterprises Inc.	Both hands. Three trials. Adjusted to hand size. 20–30 s min recovery. Higher value. Familiarization.	Dominant CHD: 11.96 ± 4.18 (kPa) non-CHD: 15.18 ± 4.44 (kPa) Non-Dominant CHD: 14.29 ± 5.14 (kPa) non-CHD: 15.18 ± 4.85 (kPa)	High
**Adolescents (age 12 to ≤ 18)**									
Izquierdo-Gomez, 2013[44]	Cross-sectional	111	DS: 15.47 ± 2.03 non-DS: 13.90 ± 0.96	DS: Female: 5 Male:12 non-DS: Female: 45 Male: 49	DS: 22.6 non-DS: 21.7	TKK 5101 Grip D.	Dominant hand. Twice trials. Seated Position. Shoulder adducted and neutrally rotated. Elbow in full extension. Wrist neutral. Adjusted to hand size. * Verbal encouragement. 2 s maintenance. Short rest Higher value.	DS: 14.97 ± 5.43 Non-DS: 26.29 ± 6.63	Medium
John, 2016 [45]	Cross-sectional	60	DS: 12.3 ± 3.6 non-DS: 12.6 ± 3.4	DS: Female: 12 Male:18 non-DS: Female: 12 Male: 18	DS: 20.4 non-DS: 21.3	Jamar.	Both hands. Three trials. Seated position. Shoulder adducted and neutrally rotated. Elbow in a 90° flexion. Wrist 0° and 30° dorsiflexion and 0° and 15° ulnar deviation. Adjusted to hand size. Verbal encouragement. Higher value.	Dominant DS: 6.3 ± 5.1 non-DS: 15.9 ± 5.1 non- Dominant: DS: 5.8 ± 4.9 non-DS: 14.9 ± 5.4	Higher
Matute-Llorente, 2017[52]	Cross-sectional	42	DS: 15,5 ± 3,6	DS: Female: 12 Male:18	–	TKK 5101 Grip D.	Dominant hand. Twice for each hand. Stand Position. Shoulder adducted and neutrally rotated. Elbow in full extension. Wrist neutral. Adjusted to hand size.	1 cm below: 14,9 Optimal grip spam: 15,3 1 cm above:15,2	Higher
Melo, 2022 [11]	Cross-sectional	56	DS: 12.46 ± 2.88 Female: 12.64 ± 3.04 Male: 12.23 ± 2.77 non-DS: 12.38 ± 3.07 Female: 12.29 ± 3.4 Male: 12.55 ± 2.50	DS: Female: 17 Male:13 non-DS: Female: 17 Male: 9	DS: 21.74 Female: 22.00 Male: 21.39 non-DS: 20.09 Female: 20.38 Male: 19.55	Jamar.	Dominant hand. Three trials. Seated position. Shoulder adducted and neutrally rotated. Elbow in a 90° flexion. Wrist 0° and 30° dorsiflexion and 0° and 15° ulnar deviation. Adjusted to hand size. Verbal encouragement. 5 s maintenance. 1 min recovery. Higher value. Familiarization.	DS: 10.43 ± 6.72 Female: 8.88 ± 4.74 Male:12.46 ± 8.45 non-DS: 18.36 ± 7.84 Female: 16.91 ± 6.4 Male: 21.11 ± 0.85	Low
**Adolescents (age 12 to ≤ 18)**									
Pessoa, 2023 [46]	Longitudinal	16	15.22 ± 2.44	Female: 7 Male: 9	Pre: 28.43 Pos: 28.53	TKK 5101 grip D.	Both hands. Twice for each hand. Seated position. Shoulder adducted and neutrally rotated. Elbow in full extension. 2 s maintenance. Average value.	Pre: 8.60 ± 3.95 Pos: 10.67 ± 4.28	Low
Pino-Valenzuela, 2023[47]	Longitudinal	16	2009: 12.5 2014: 17.5 2019: 22.5	Female: 3 Male: 13	2009: 21.5 2014: 24.4 2019: 26.7	Baseline, model 12-086.	Both hands. Twice trials. Stand position. Shoulder neutrally rotated. Elbow in full extension. 3- 5 s maintenance. Higher value. Familiarization.	Dominant 2009: 19.1 ± 4.4 2014: 21.7 ± 3.4 2019: 21.9 ± 3.2 Non-Dominant 2009: 17.7 ± 4.5 2014: 19.8 ± 3,7 2019: 19.9 ± 3,8	Low
Suarez-Villadat, 2019 [49]	Longitudinal	263	DS 2012: 15.7 ± 2.4 2013: 16.7 ± 2.4 2014: 17.6 ± 2.5 non-DS 2012: 13.8 ± 1.4 2013: 14.7 ± 1.4 2014: 15.7 ± 1.4	DS: Female: 36 Male:64 non-DS: Female: 55 Male: 108	DS 2012: 23.6 2013: 24.3 2014: 24.8 non-DS 2012: 20.9 2013: 21.4 2014: 21.8	TKK 5101, Grip D.	Dominant hand. Twice trials. Seated and stand position. Shoulder adducted and neutrally rotated. Elbow in full extension. Wrist neutral. Adjusted to hand size. * Verbal encouragement. 2 s maintenance. Short rest. Higher value.	DS 2012: 15.9 ± 6.6 2013: 17.4 ± 5.9 2014: 17.9 ± 6.9 non-DS 2012: 26.5 ± 7.7 2013: 30.7 ± 7.8 2014: 33.9 ± 7.9	Low
Tejero-Gonzalez, 2013[12]	Cross-sectional	17	15.4 ± 2.0	Female: 5 Male: 12	Test:22.34 Retest: 22.18	TKK 5101 Grip D.	Both hands. Twice trials. Seated position. Shoulder adducted and neutrally rotated. Elbow in full extension. Wrist neutral. Adjusted to hand size. * Verbal encouragement. 2 s maintenance. Short rest. Higher value.	Test: 15.71 ± 6.42 Retest: 15.59 ± 6.52	High
**Adults (age 18 >)**									
Balic, 2000 [33]	Cross-sectional	20	SO: 23.4 ± 3.7 Sedentary group: 25.9 ± 2.5	SO: Female: 4 Male:9 Sedentary group: Female: 2 Male:5	SO: 25.7 Sedentary group: 28.1	Takei-Kiki	Both hands. Measured twice. Verbal encouragement. Higher value. Familiarization.	Dominant: SO: 29.9 ± 8.5 Sedentary group: 23.0 ± 8.9 Non-Dominant SO: 29.1 ± 8.1 Sedentary group: 25.6 ± 7.1	High
Bunsawat, 2016 [39]	Cross-sectional	18	DS: 26.0 ± 3 non-DS: 28.0 ± 3	DS: Female: 4 Male:6 non-DS: Female: 6 Male: 2	DS: 29.8 non-DS: 26	Biopac Systems Inc.	Dominant hand. Three trials. Seated position Shoulder adducted and neutrally rotated. Elbow in a 90° flexion. Higher value. Familiarization.	DS: 15.3 ± 1.3 Non-DS: 19.7 ± 2.1	High
Cabeza-Ruiz, 2009[50]	Cross-sectional	22	Female: 24.88 ± 5.22 Male:27.79 ± 6.34	Female: 8 Male: 14	–	Ergometer model from Globus.	Both hands. Three trials for each hand. Stand position. Shoulder adducted and neutrally rotated. Elbow in full extension. Neutral wrist position. 6 s maintenance. Higher value.	Dominant: Female: 18.52 ± 5.28 Male: 29.18 ± 11.79 Non-Dominant: Female: 19.42 ± 4.93 Male: 29.72 ± 12.33	High
Cabeza-Ruiz, 2019 [13]	Cross-sectional	37	37.57	Female: 11 Male: 26	Test: 30.75 Retest: 30.58	Jamar.	Both hands. Twice for each hand. Stand position. Shoulder adducted and neutrally rotated. Elbow in full extension. Neutral wrist position. Adjusted to hand size. Verbal encouragement. 2 s maintenance. 10 s recovery. The average of the total scores in both hands. Familiarization.	Test: 21.41 ± 6.25 Retest: 21.54 ± 6.67	Medium
**Adults (age 18 >)**									
Chen, 2018 [36]	Cross-sectional	14	21.54		33.91	Jamar.	The average of the total scores in both hands Three trials for each hand. Stand position. Shoulder adducted. Elbow in a 90° flexion. Wrist 0° and 30° dorsiflexion and 0° and 15° ulnar deviation. Hand-adjustable in second position. Verbal encouragement. 5 s maintenance. 15 s recovery.	22.50 ± 7.45	High
Chen, 2014 [37]	Cross-sectional	20	Exercise group: 21.76 ± 4.79 Control group: 17.77 ± 3.49	Male: 20	Exercise group: 32.08 Control group: 33.12	Jamar.	The average of the total scores in both hands Three trials for each hand. Stand position. Shoulder adducted and neutrally rotated. Elbow in a 90° flexion. Wrist 0° and 30° dorsiflexion and 0° and 15° ulnar deviation Hand-adjustable in second position. Verbal encouragement. 5 s maintenance. 15 s recovery.	Exercise group: Pre-test: 49.69 Pos-test: 49.95 Control group: Pre-test: 42.71 Pos-test: 39.70	High
Chen, 2019 [35]	Cross-sectional	28	High-Intensity exercise: 22.30 Low-Intensity exercise: 21.74 Control: 20.23		High-Intensity exercise: 29.69 Low-Intensity exercise: 32.87 Control: 35.68	Jamar.	The average of the total scores in both hands. Three trials for each hand. Stand position. Shoulder adducted and neutrally rotated. Elbow in a 90° flexion. Wrist 0° and 30° dorsiflexion and 0° and 15° ulnar deviation. Hand-adjustable in second position. Verbal encouragement. 5 s maintenance. 15 s recovery.	High-Intensity exercise: 38.29 ± 7.35 Low-Intensity exercise: 51.33 ± 14.01 Control: 42.54 ± 11.30	High
Chen, 2021 [34]	Cross-sectional	55	Young group: 21.37 ± 5.14 Older group: 39.74 ± 8.96	Female: 21 Male: 34	Young group: 32.54 Older group: 33.08	Jamar.	The average of the total scores in both hands. Three trials for each hand. Stand Position. Shoulder adducted and neutrally rotated. Elbow in a 90° flexion. Wrist 0° and 30° dorsiflexion and 0° and 15° ulnar deviation. Hand-adjustable in second position. Verbal encouragement. 5 s maintenance. 15 s recovery.	Young group: 20.55 ± 7.13 Older group: 20.81 ± 7.98	High
**Adults (age 18 >)**									
Coelho-Junior, 2019 [5]	Cross-sectional	105	DS: 38.4 ± 12.1	Female: 61 Male: 43	27.9	Jamar.	Dominant hand. One trial. Seated Position. Shoulder adducted and neutrally rotated. Elbow in a 90° flexion. Wrist neutral. Verbal encouragement. 4 s recovery. Higher value. Familiarization.	12.6 ± 6.0	Medium
Fedrigo, 2023 [51]	Cross-sectional	26	Female: 30.7 ± 10.3 Male: 23.3 ± 5.1	Female: 18 Male: 8	Median = 24.8 (IQR =23.5–29.5)	Jamar.	Both hands. Three trials for each hand. Seated position Elbow in a 90° flexion. Higher value.	Dominant: Female: Median =30.0 Male: Median = 30.0 Non-Dominant: Female: Median 21.0 Male: Median 20.0	Medium
Fernhall, 2003 [42]	Cross-sectional	24	DS: 23.8 ± 1.8 non-DS: 26.4 ± 1.0	DS: Female: 6 Male:6 non-DS: Female: 6 Male: 6	DS: 34.8 non-DS: 22.8	TSD121C	Dominant hand. Three trials. Supine Position. Verbal encouragement. 2 s recovery. Higher value. Familiarization.	DS: 10.9 ± 1.4 Non-DS: 26.6 ± 2.4	Medium
Figueroa, 2005 [41]	Cross-sectional	27	DS: 27.8 non-DS: 26.4		DS: 32.3 non-DS: 25.9	BioPac Systems Inc.	Dominant hand. Three trials. Seated Position. Elbow in a 90° flexion. Adjusted to hand size. Higher value. Familiarization.	DS: 12.1 ± 5.1 non-DS: 26.6 ± 8.1	Medium
**Adults (age 18 >)**									
Godoy, 2005 [38]	Cross-sectional	138	DS: 27.82 ± 6.63 non-DS: 27.92 ± 6.17	DS: Female: 14 Male: 14 non-DS: Female: 55 Male: 55	–	Jamar.	Both hands. Three trials for each hand. Seated position. Shoulder adducted and neutrally rotated. Elbow in a 90° flexion. Wrist 0° and 30° dorsiflexion and 0° and 15° ulnar deviation. Hand-adjustable in second position. Verbal encouragement. 5 s maintenance. 1 min recovery. Higher value.	Dominant: DS: 18.25 ± 5.19 non-DS: 36.76 ± 10.63 non-Dominant: DS: 17.56 ± 5.00 non-DS: 33.01 ± 10.32	Medium
Heffernan, 2005 [40]	Cross-sectional	22	DS: 25.0 ± 2.4 non-DS: 27.5 ± 2.7	DS: Female: 5 Male:7 non-DS: Female: 5 Male: 5	DS: 29.6 non-DS: 26.6	TSD121C, BioPac Systems.	Dominant hand. Three trials. Supine position. Verbal encouragement. 2 s maintenance. Higher value. Familiarization.	DS: 11.6 ± 1.6 Non-DS: 27.4 ± 2.8	High
Heffernan, 2009 [43]	Cross-sectional	86	DS: 26.3 ± 1.5 Young non-DS: 26.7 ± 1.3 Older non-DS: 70.1 ± 1.0	DS: Female: 16 Male: 13 Young non-DS: Female: 11 Male: 13 Older non-DS: Female: 23 Male: 10	DS: 30.3 Young non-DS: 24.6 Older non-DS: 27.9	TSD121C, BioPac Systems.	Dominant hand. Three trials. Seated Position. Elbow in a 90° flexion. Wrist neutral. Adjusted to hand size. Verbal encouragement. Higher value. Familiarization.	DS: 36.7 ± 2.7 Young non-DS: 60.2 ± 6.2 Older non-DS: 33.5 ± 2.0	High
Rosety-Rodrigues, 2021 [48]	Longitudinal	36	Experimental: 28.4 Control: 27.8	–	Experimental: 31.4 Control: 30.8	Jamar.	Dominant hand. Three trials. Seated position. Shoulder adducted and neutrally rotated. Wrist neutral. Hand-adjustable in first position. Verbal encouragement. 5 s maintenance. 1 min recovery. Higher value. Familiarization.	Pre: 28.3 ± 7.2 Pos: 30.8 ± 7.4	Low
**Adults (age 18 >)**									
Terblanche, Boer, 2013 [32]	Cross-sectional	371	Female age groups: 18–25: 21.7 ± 2.2 26–35: 30.8 ± 3.0 36–45: 40.1 ± 3.0 >45: 51.5 ± 5.3 Male age groups: 18–25: 21.4 ± 2.4 26–35: 30.5 ± 3.1 36–45:40.3 ± 3.0 >45: 50.7 ± 3.8	Female 18–25: 46 26–35: 52 36–45: 46 >45: 28 Male 18–25: 53 26–35: 58 36–45:58 >45: 30	Female 18–25: 32.0 26–35: 31.4 36–45: 33.1 >45: 31.1 Male 18–25: 30.8 26–35: 30.1 36–45:28.9 >45: 28.2	TKK 5401; Grip D.	Dominant hand. Three trials. Seated position. Adjusted to hand size. Verbal encouragement. 30 s maintenance. Higher value.	Female age groups: 18–25: 20.7 ± 4.8 26–35: 21.8 ± 5.5 36–45:20.9 ± 5.6 >45:18.4 ± 6.2 Male age groups: 18–25: 30.1 ± 8.1 26–35: 30.6 ± 9.3 36–45: 29.3 ± 8.2 >45: 29.0 ± 8.0	High

DS: Down syndrome; BMI: body mass index; CHD: congenital heart disease; HGS: HGS; SO: Special Olympics; Kpa: Kilo pascal.

### Risk of bias and quality of evidence

The risk of bias and study quality was assessed individually by two reviewers (GLRM and RCES) using the Newcastle-Ottawa Scale, which evaluate three domains: selection, comparability, and exposure/outcome [29]. Each study was considered to have a low, medium or high risk of bias (Table 1 and Supplementary Table 5).

### Statistical analyses

All analyses were conducted using R, version 4.3.2. A meta-analysis assessed the association between HGS values and the number of controlled variables in HGS measurement protocols, using a random-effects model to estimate proportions and 95% confidence intervals (CI). Heterogeneity was evaluated with Cochran’s Q test and I^2^, with thresholds for low, moderate, and high inconsistencies set at 25%, 50%, and 75%. Significance was set at *p* < 0.05. A spline regression model, adjusted for heterogeneity and cohort size, analyzed the relationship between HGS values and controlled variables, using knots at the 10th, 50th, and 90th percentiles [30]. The Wald test assessed linearity, and no better fit was found with linear. Subgroup analysis considered group (DS or non-DS), population (children, adolescents, and adults), and BMI (healthy, overweight, obese). Studies lacking these details were excluded. Bayesian multilevel regression analyzed individual variables, with HGS values reported based on the presence or absence of each variable, with 95% confidence intervals. Bayesian multilevel regression was used to analyze individual protocol measurement variables, with all variables entered into the model. The conditional effects represented HGS values according to the presence or absence of a variable (i.e. 0 if not reported, 1 if reported) [31]. Estimates were reported with 95% confidence intervals.

## Results

### Study selection

A total of 441 potentially eligible studies were retrieved from six databases. Moreover, 3 additional records were identified using other sources. A total of 232 duplicate studies were excluded, and the remaining 161 records excluded based on title/abstract view. The remaining 51 full-text studies were retrieved for complete review. Another 22 studies were excluded because they did not meet the eligibility criteria. Finally, 29 studies remained in the final selection for the systematic review [5,11–13,25–28,32–52]. The flowchart of the study selection process is shown (Figure 1).

### Study characteristics

Studies included in this systematic review were published between 1982 and 2023. Twenty-five were cross-sectional studies and four were longitudinal studies. A total of 1816 participants (42.23% female) were enrolled, with participant ages ranging from 2 [28] to 51 years [32]. The sample sizes ranged from 14 participants [36] to 371 [32] (Table 1). Only two studies have evaluated the reliability and validity of HGS protocols in individuals with DS. Tejero-Gonzalez et al. [12] reported ICCs of 0.86 (0.64–0.95) for adolescents, while Cabeza-Ruiz et al. [13] found an ICC of 0.9 (0.81–0.95) for adults.

### Description of HGS measurement

Some studies provided limited information on the protocols used. As shown in Table 1, all included studies controlled the type of dynamometer used. However, only 13 articles carried out familiarization, and 14 articles calibrated the dynamometer. The Jamar dynamometer was the most frequently used (*n* = 13), followed by the Takei Kiki dynamometer (*n* = 7). Twenty-six studies (89%) described the individual’s posture, with the majority measured in a seated position (*n* = 15), 8 in a standing position, and 2 in a supine position. One study reported variations in posture based on individual ability. Fifteen studies measured HGS in dominant and non-dominant hands (52%), and 14 measured only the dominant hand (48%). Nineteen studies reported shoulder position (65%), 24 reported elbow position (82%), and 19 (82%) reported wrist position. The dynamometer handle was mentioned in 27 articles, with the second handle position mentioned in 16 articles (55%). Sixty-two percent controlled handgrip duration, 55% reported the recovery time, 58% used the higher value estimation for the analysis, and 75% reported whether verbal encouragement was given during measurement (Table 1). The ASHT protocol was mentioned in 18 studies, while 11 studies did not specify the protocol used. Table 2 summarizes the standard protocol for measuring HGS based on the most common characteristics reported in the studies.

**Table 2.** Summary of the standard measurement protocol for the measurement of HGS.

Protocol items	Characteristics
Type of dynamometer	Jamar dynamometer.
Laterality of the tested hand	Both hands.
Body position	Seated position (use the same chair for each measurement).
Arm position	Shoulder in adduction and neutral rotation.
Elbow position	Flexion to 90°, forearm in neutral position.
Wrist position	15–30° of extension and 0–15° of ulnar deviation.
Hand-adjustment	Second handle position.
Number of measurements	Three trials.
Duration of grip	At least 5s.
Value estimation	Higher value.
Recovery time	At least 60s.
Verbal encouragement	Yes.

To design the summary of the HGS measurement protocol, we considered the most frequent characteristic for each item in the included studies. If a variable was poorly defined in the included studies, the item was described based on previous protocols (American Society of Hand Therapists protocol [19] or Southampton protocol [17]).

## Quantitative analysis

Eleven of the 29 studies were included in the meta-regression analysis, with a total of 656 participants with and without DS [11,26,27,38–45]. The random-effects model indicated that the number of reported variables in the HGS protocol was significantly associated with an increase in HGS, with a mean estimate of 20.59 units (SE = 2.59, *p* < 0.0001, 95% CI = [15.49 − 25.68]). High heterogeneity was observed (I^2^ = 94.33%). A mixed-effects meta-regression assessed the influence of moderators (group, age, and BMI) on HGS, revealing that although HGS varied across these factors, none showed statistically significant effects. The model accounted for 23.87% of the heterogeneity (R^2^ = 23.87%), but a large portion remained unexplained (I^2^ = 93.49%, tau^2^ = 114.89, *p* < 0.0001). The intercept for adolescents with a healthy weight and DS was significant (16.82, *p* = 0.0107), and age had a marginal effect, with adults showing HGS 14.55 units higher than other groups (*p* = 0.0627). Individuals without DS had an average HGS 6.89 units higher (*p* = 0.3941), while obesity and overweight were linked to lower HGS, although not statistically significant (*p* = 0.1948 and *p* = 0.1404, respectively) (Supplementary Table 2).

**Figure 2.**
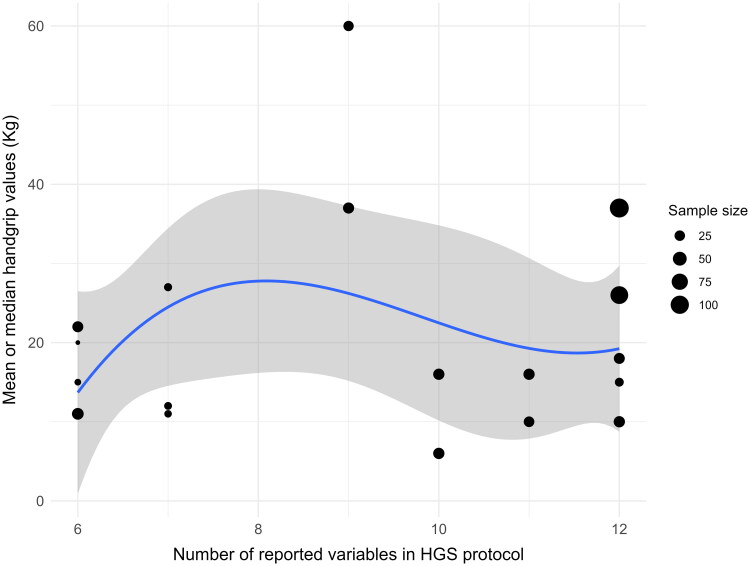
Association of the number of variables controlled in the handgrip measurement protocols and HGS values. The solid curve indicates the non-linear trend for the association between the number of variables controlled in the protocol and the mean or median handgrip values (kg) and its corresponding 95% confidence interval based on a spline model with three knots at the 10th, 50th and 90th percentile of the pooled HGS. The area of each data point is proportional to each study’s sample size.

**Figure 3.**
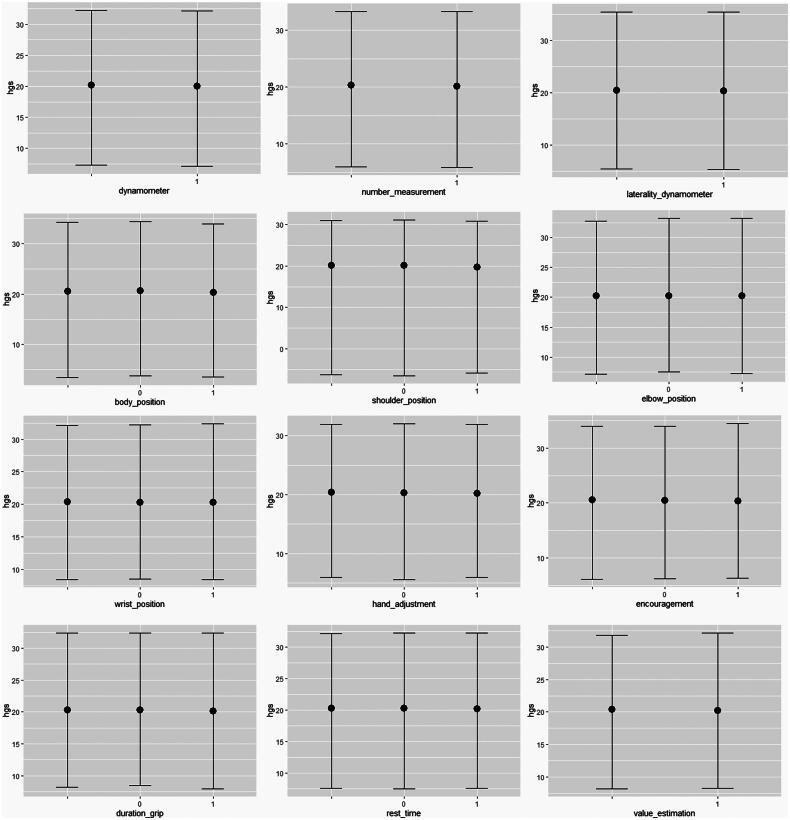
Multilevel Bayesian regression for the association between individual protocol variables and HGS values (kg). Value 0 indicates that the variable was not reported and value 1 indicates that the variable was reported. Estimates are presented with 95% confidence interval.

The spline regression analysis revealed an intercept of 13.4231 (SE = 6.03, *p* = 0.03). The slopes for the number of reported variables were: (i) between 6 and 7 variables = 0.6806; (ii) between 7 and 9 variables = 25.2859; (iii) between 9 and 12 variables = −10.6920 (Figure 2). The model explained 82.66% of the variation in HGS, indicating a good fit (F [8, 9] = 5.362, *p* = 0.01). The maximum HGS is reached at point 8 of the protocol due to the presence of adults in the sample. The findings highlighted that the number of reported variables positively affects HGS, adults had 47.61 units higher HGS than children (*p* = 0.0009), and obesity was associated with a decrease of 15.68 units (*p* = 0.0675). Sample size and group showed no significant effects (Figure 3 and Table 3).

**Table 3.** Spline regression modeling to evaluate the factors that influence handgrip strength.

Variables	Estimate	SE	*t* value	*p* value
Intercept	−85.59	24.50	−3.49	**0.006**
Number variables in HGS protocol	8.24	2.11	3.89	**0.003**
Sample_size	1.90	1.20	1.58	0.147
Group (non-DS)	1.45	6.41	0.22	0.825
Cat_age (Adults)	47.61	9.82	4.84	**0.0009**
Cat_age (Children)	36.63	17.83	2.05	0.07
BMI (obesity)	−15.68	7.54	−2.07	0.06
BMI (overweight)	−1.98	5.31	−0.37	0.71

DS: Down syndrome; BMI: Body mass index; SE: Standard Error.

**Figure 4.**
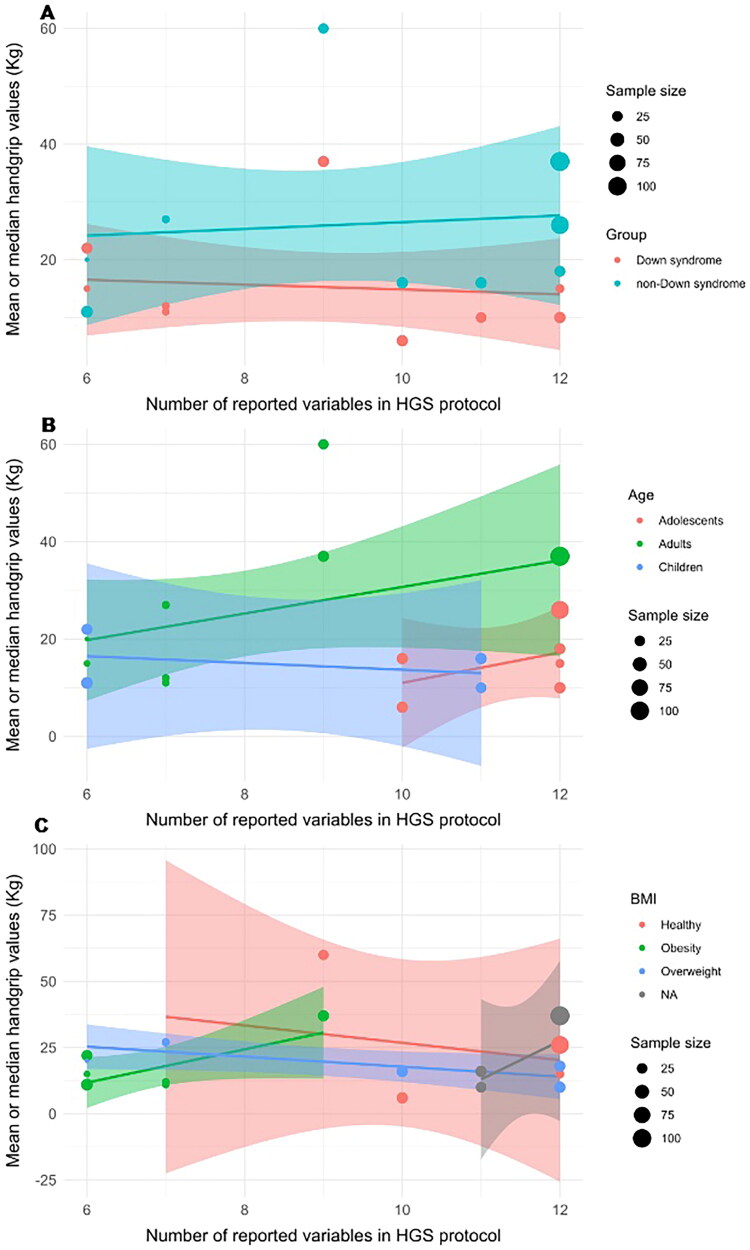
Association of the number of controlled variables in the protocols and HGS values stratified by group (a), age (B), and body mass index (BMI) (C). BMI, body mass index; N/A; studies did not report BMI values [27,38]. The curve indicates the trend of the association for each category and its corresponding 95% confidence interval. The area of each data point is proportional to each study’s sample size.

The results of the multilevel Bayesian regression for the binary outcomes of each variable from the HGS measurement protocol are summarized in Figure 4, with estimates presented in Supplementary Tables 3 and 4. The findings indicate that the variables in the HGS protocol do not have a significant impact on HGS values. The variation between groups is moderate, with standard deviations ranging from 7.62 kg to 8.74 kg, but there is considerable uncertainty in these estimates, as indicated by the wide credibility intervals.

## Risk of bias assessment

Of the 29 included studies, quality assessment using the Newcastle-Ottawa Scale indicated that 41.66% of cross-sectional studies had a low or medium risk of bias, and all longitudinal studies had a low risk of bias (Table 1 and Supplementary Table 5).

## Discussion

This is the first systematic review to analyze the measurement protocols used to assess HGS among individuals with DS. The methodologies employed for HGS measurements demonstrated considerable variation and heterogeneity across the reviewed studies. For instance, the number of repetitions, recovery time between repetitions, and the provision of verbal encouragement varied among the identified protocols. Furthermore, some studies did not control for critical aspects of the protocol, such as shoulder position, elbow position, and hand adjustment to the dynamometer. Conversely, value estimation, laterality of the tested hand, number of measurements, and type of dynamometer were consistently included in all studies reviewed.

Our meta-regression analysis revealed that HGS values were generally higher in studies that reported a greater number of variables in the standard measurement protocol (Figure 3). However, this relationship was nonlinear; studies reporting more than eight variables showed a decrease in HGS. While age influenced HGS values, factors such as sample size, BMI, and group did not demonstrate significant effects. Unfortunately, a sex subgroup analysis was not conducted, as the studies did not provide HGS values separately for individuals with and without DS. Nevertheless, interpretations of analyses stratified by subgroups should be approached with caution due to the inclusion of fewer studies, rendering them more unstable.

Regarding the type of dynamometer, this review demonstrated the predominance of the use of Jamar, with Takei being the second most used. However, previous reviews have shown low agreement between the Jamar and Takei dynamometers [53], while alternatives such as the Baseline and pneumatic bulb dynamometers were found to be valid and comparable to the Jamar dynamometer [54]. The Jamar hydraulic dynamometer is reliable for individuals with DS [12,13].

Furthermore, it was observed that when the reviewed studies controlled for the adjustment of the hand to the dynamometer, it did not significantly affect the values of HGS. However, ASHT recommends using a Jamar dynamometer calibrated to the second handle position to measure HGS [19], while the Physical Activity Level (ALPHA) health-related physical fitness test suggests adjusting the handgrip handle according to hand size [55]. Our review revealed that 70% of the included studies reported this hand size information (Table 1).

The reviewed studies predominantly used the seated position for assessing HGS, although consistency was observed between supine and seated positions when upper limb positions were identical [56]. This could benefit assessments of individuals with DS, who may experience balance issues or muscle hypotonia [2]. While ASHT protocols recommend specific upper extremity positions [19], our review found limited reporting of elbow and shoulder positions. Bayesian regression analysis suggested that reporting shoulder position does not significantly affect the values of HGS.

Regarding hand laterality, HGS typically favors the dominant side, but differences may vary depending on individual dominance [57]. An accurate assessment of dominance and laterality is crucial as both factors influence HGS [34,57]. All studies in the review reported the laterality of the tested hand. Concerning about recovery time, most studies suggested longer recovery times (e.g. 60 s rest) to mitigate muscle fatigue, ASHT recommended at least 15 s [19]. Studies opted for longer recovery times due to characteristics of individuals with DS, who exhibit higher exercise intolerance and fatigue due to mitochondrial dysfunction [58,59]. Therefore, longer recovery times are recommended to enhance the effectiveness of the test [58,60].

Differences in HGS measurement protocols, such as the number of measurements (one/two/three trials) and value estimation (higher/average value) are used to determine maximal HGS, and could impact measurement reproducibility and comparability across populations with and without ID [61]. While some studies recommend the mean of three trials [19,62], others suggest using the maximum HGS score from six trials [16]. However, using the mean of three trials may provide a more accurate measure for individuals with ID [61]. Our study highlights the importance of standardizing measurement protocols, particularly given the high heterogeneity observed in previous literature review [18].

The literature indicates that individuals with DS typically have muscle strength about half as much as the general population [4,60,63]. A recent study found HGS values in the DS group to be 13.96 kg lower than in the non-DS group [64]. Despite lower HGS values observed in individuals with DS, longitudinal studies have underscored the importance of annual assessments to monitor their musculoskeletal fitness [47,49]. However, caution is advised in interpreting these results due to potential measurement bias observed across most of HGS protocol variables. Previous study suggest familiarization over one to three non-consecutive days to improve DS individuals’ understanding of assessment tools [60,65]. Notably, only 44.8% of the studies in this review implemented familiarization.

In addition, previous studies have emphasized the importance of integrating verbal and visual encouragement to ensure maximum effort during a task [20,21]. The utilization of verbal encouragement and visual feedback resulted in approximately 9.7% and 7.7% higher HGS, respectively, compared to non-use [21]. Our review found that 75% of the included studies reported verbal encouragement (Table 1). However, it is essential to standardize verbal commands by using simple and direct language, and providing visual demonstrations of movement execution for individuals with DS [22,23]. Verbal encouragement should not be loud or akin to shouts (e.g. ‘go, go, now’), as this may not motivate individuals with DS, but instead deter them. Boato et al. [65] illustrated the significance of incorporating Henri Wallon’s emotion theory and considering emotional expressions in a laboratory assessment setting, allowing adolescents with DS to comprehend how to perform the HGS test through visual feedback and verbal encouragement.

To reinforce verbal-motor learning, the literature has underscored the significance of visual feedback, particularly as individuals with DS encounter challenges in processing information and making decisions solely through verbal commands [66]. Additionally, a previous study has highlighted the principle of intrinsic reward, whereby the assessment environment must be considered, including factors such as peer assessment, the presence of a family member, or the option to choose music to listen to during the session, as practical teaching strategies to facilitate the task [67].

### Strengths and limitations

This systematic review included data from more than 1.816 individuals with and without DS across a wide age range, including 1.201 with DS. Our study is the first to examine HGS measurement protocols in individuals with DS. The present study has several limitations. Although we systematically searched six relevant databases, it is still possible that some studies were included in this review. Studies that used languages other than English, Spanish, or Portuguese were included in the final selection.

Furthermore, as the quality of studies is often linked to a more detailed reporting of HGS measurement protocols, it is unlikely that the gray literature (i.e. studies not indexed in the databases examined) will provide protocol information with more details than the selected studies. Importantly, although we acknowledge that reporting and publication bias cannot be entirely dismissed, we assume that the information reported in the protocol studies is reliable. Finally, information on HGS measurement protocols could not be analyzed in a meta-regression analysis of the 18 studies.

## Conclusion

In summary, HGS assessment protocols for individuals with DS tend to be heterogeneous. To enhance accuracy, it is essential to follow standardized measurement protocols and control key variables, as this has been linked to higher HGS values. Considering participant characteristics such as age, BMI, and group is also important. Familiarity with the assessment tool improves test comprehension, while incorporating verbal and visual encouragement can significantly boost engagement and performance during the assessments.

## PROSPERO registry number

CRD42023485653.

## Supplementary Material

Supplementary.docx

## Data Availability

Data from this study are available from the corresponding author upon reasonable request.
