# Supplementary material for: Handgrip strength measurement protocols in individuals with Down syndrome: a systematic review and meta-regression: Handgrip strength measurement protocols and Down syndrome
Source: Ann Med. 2025 Jan 22;57(1):2453077. doi: 10.1080/07853890.2025.2453077 (PMC11755743; doi:10.1080/07853890.2025.2453077)
Supplement: Supplementary.docx [file IANN_A_2453077_SM4662.docx]

**Supplementary material**

**Search Strategy and terms and additional inclusion and exclusion criteria.**

PICOS

*Participants:* Individuals with DS, and study samples that explicitly include individuals with DS and report their outcomes separately, regardless of age.

*Intervention:* Not applicable as we are not evaluating the effects of interventions.

*Comparator:* Studies with or without a comparator group were included.

*Outcome:* The outcome measures described were type of dynamometer for the assessment of HGS, individual’s position (including shoulder, elbow, arm, and handle and posture), hand dominance, number of repetitions, acquisition, rest time, encouragement, familiarization, employing either single or repetitive measures on handgrip strength and handgrip strength values (mean absolute).

*Study type:* Cross-sectional and longitudinal studies.

**Supplementary table 1. Search strategy**

| Database: PubMed up until December 2023 | | |
| --- | --- | --- |
| # | Searches | Results |
| 1 | **(("Down syndrome" OR "trisomy 21") AND ("hand" OR "handgrip" OR "grip" OR "grasp" OR "dynamometer" OR "hand grip")) AND ("force" OR "strength" OR "isometric" OR "strength test" OR "strength measure" OR "muscular test")** | 70 |

| Database: Web of Science (WOS) up until December 2023 | | |
| --- | --- | --- |
| # | Searches | Results |
| 1 | ((ALL= ("Down syndrome" OR "trisomy" OR "trisomy 21”)) AND ALL= ("hand" OR "handgrip" OR "grip" OR "grasp" OR "dynamometer" OR "hand grip")) AND ALL= ("force" OR "strength" OR "isometric" OR "strength test" OR "strength measure" OR "muscular test") | 86 |

| Database: Scopus up until December 2023 | | |
| --- | --- | --- |
| # | Searches | Results |
| 1 | TITLE-ABS-KEY "Down syndrome" OR "trisomy 21" AND "hand" OR "handgrip" OR "grip" OR "grasp" OR "dynamometer" OR "hand grip" AND "force" OR "strength" OR "isometric" OR "strength test" OR "strength measure" OR "muscular test" | 128 |

| Database: Embase up until December 2023 | | |
| --- | --- | --- |
| # | Searches | Results |
| 1 | Down syndrome AND Hand grip | 33 |

| Database: OVID up until December 2023 | | |
| --- | --- | --- |
| # | Searches | Results |
| 1 | "Down syndrome" OR "trisomy 21" AND "hand" OR "handgrip" OR "grip" OR "grasp" OR "dynamometer" OR "hand grip" AND "force" OR "strength" OR "isometric" OR "strength test" OR "strength measure" OR "muscular test" | 71 |

| Database: ERIC up until December 2023 | | |
| --- | --- | --- |
| # | Searches | Results |
| 1 | Down syndrome AND Hand grip | 53 |

**Supplementary table 2. Mixed Effects Model Results**

| **Variables** | **Estimate** | **SE** | **Z value** | **P value** | **95% CI** |
| --- | --- | --- | --- | --- | --- |
| Intercept | 16.82 | 6.59 | 2.55 | **0.01** | (3.90 – 29.74) |
| Group (non-DS) | 6.89 | 8.08 | 0.85 | 0.39 | (-8.96 - 22.74) |
| Cat_age (Adults) | 14.55 | 7.81 | 1.86 | 0.06 | (-0.76 – 29.86) |
| Cat_age (Children) | 10.27 | 13.92 | 0.73 | 0.46 | (-17.01 – 37.55) |
| BMI (obesity) | -14.03 | 10.82 | -1.29 | 0.19 | (-35.26 – 7.18) |
| BMI (overweight) | -10.24 | 6.95 | -1.47 | 0.14 | (-23.87 – 3.37) |

*Note:* DS, Down syndrome; BMI, Body mass index; SE, Standard Error; CI, Confidence Interval.

**Supplementary table 3. Conditional effects of handgrip variables**

| **Number of reported variables in HGS protocols** | **Estimate** | **SE** | **95% CI** |
| --- | --- | --- | --- |
| **Dynamometer (intercept)** | 20.17 | 5.92 | (7.32 – 32.29) |
| dyn = 1 | -0.14 | 0.99 | (-2.10 – 1.79) |
| group-level effects (intercept) | 7.62 | 5.92 | (0.43 – 22.89) |
| **Laterality_dynamometer (intercept)** | 20.44 | 6.73 | (5.41 – 35.38) |
| lateral = 1 | -0.12 | 1.00 | (-2.12 – 1.84) |
| group-level effects (intercept) | 8.28 | 6.49 | (0.41 – 25.55) |
| **Body_position (intercept)** | 20.22 | 6.89 | (3.48 – 34.19) |
| body = 0 | 0.03 | 1.01 | (-1.94 – 2.03) |
| body = 1 | -0.17 | 1.00 | (-2.11 – 1.83) |
| group-level effects (intercept) | 8.74 | 6.33 | (0.73 – 24.53) |
| **Shoulder_position (intercept)** | 18.74 | 7.95 | (0.74 – 26.03) |
| shoulder = 0 | 0.08 | 1.01 | (-1.98 – 2.01) |
| shoulder = 1 | -0.15 | 0.96 | (-2.09 – 1.73) |
| group-level effects (intercept) | 8.74 | 6.65 | (0.74 – 26.03) |
| **Elbow (intercept)** | 20.22 | 5.93 | (7.14 – 32.75) |
| elbow = 0 | 0.00 | 0.97 | (-1.84 – 1.84) |
| elbow = 1 | -0.08 | 0.98 | (-1.98 – 1.83) |
| group-level effects (intercept) | 8.16 | 6.49 | (0.43 – 24.61) |
| **Wrist_position (intercept)** | 20.32 | 5.67 | (8.44 – 32.09) |
| wrist = 0 | -0.07 | 0.99 | (-1.99 – 1.90) |
| wrist = 1 | -0.02 | 0.98 | (-1.87 – 1.91) |
| group-level effects (intercept) | 7.73 | 5.88 | (0.56 – 22.22) |
| **Hand_adjustment (intercept)** | 20.04 | 6.14 | (0.58 – 26.14) |
| hand = 0 | -0.09 | 0.98 | (-1.99 – 1.82) |
| hand = 1 | -0.04 | 0.99 | (-2.02 – 1.91) |
| group-level effects (intercept) | 8.13 | 6.27 | (0.58 – 26.14) |
| **Number_measurements (intercept)** | 20.15 | 6.36 | (5.96 – 33.31) |
| number_measurements = 1 | -0.15 | 0.98 | (-2.10 – 1.81) |
| group-level effects (intercept) | 8.41 | 7.06 | (0.55 – 26.93) |
| **Duration_grip (intercept)** | 20.34 | 5.73 | (8.19 – 32.43) |
| duration = 0 | 0.03 | 1.00 | (-1.86 – 1.99) |
| duration = 1 | -0.15 | 1.00 | (-2.06 – 1.80) |
| group-level effects (intercept) | 7.74 | 5.44 | (0.58 – 21.15) |
| **Value_estimation (intercept)** | 20.34 | 5.94 | (8.15 – 31.84) |
| value_estimation = 1 | -0.12 | 1.02 | (-2.11 – 1.89) |
| group-level effects (intercept) | 8.17 | 7.06 | (0.67 – 24.02) |
| **Recovery_time (intercept)** | 20.16 | 6.02 | (7.61 – 32.20) |
| recovery_time = 0 | -0.02 | 1.01 | (-2.04 – 1.92) |
| recovery_time = 1 | -0.08 | 0.97 | (-1.96 – 1.85) |
| group-level effects (intercept) | 7.86 | 5.74 | (0.59 – 22.30) |
| **Encouragement (intercept)** | 20.40 | 6.61 | (6.08 – 33.95) |
| encouragement0 | -0.06 | 0.99 | (-2.09 -1.91) |
| encouragement1 | -0.10 | 1.02 | (-2.09 – 1.91) |
| group-level effects (intercept) | 7.87 | 5.71 | (0.66 – 22.16) |

*Note:* HGS, Handgrip strength; SE, Standard Error; CI, Confidence Interval.

| **Number of reported variables in HGS protocols** | **Down syndrome** | | | | **non-Down Syndrome** | | | |
| --- | --- | --- | --- | --- | --- | --- | --- | --- |
|  | **Estimate** | **SE** | **Q2.5** | **Q97.5** | **Estimate** | **SE** | **Q2.5** | **Q97.5** |
| Dynamometer | -3.15 | 5.96 | -16.65 | 8.93 | 3.94 | 6.07 | -7.39 | 17.84 |
| Number of measurements | -3.08 | 6.41 | -17.88 | 9.63 | 4.00 | 6.52 | -7.63 | 19.75 |
| Laterality of hand | -3.43 | 6.85 | -19.88 | 10.06 | 3.83 | 6.86 | -9.39 | 19.86 |
| Body position | -3.30 | 6.76 | -18.27 | 10.65 | 4.06 | 7.04 | -8.91 | 22.34 |
| Shoulder position | -1.94 | 7.73 | -15.59 | 21.36 | 5.60 | 8.39 | -5.83 | 32.20 |
| Elbow position | -3.27 | 6.25 | -17.50 | 8.36 | 3.92 | 6.08 | -7.43 | 17.96 |
| Wrist position | -3.25 | 5.75 | -15.98 | 7.62 | 3.65 | 5.74 | -7.01 | 16.50 |
| Hand-adjustment | -3.12 | 6.28 | -16.89 | 10.31 | 4.14 | 6.46 | -7.13 | 20.65 |
| Verbal encouragement | -3.48 | 6.69 | -18.38 | 8.71 | 3.79 | 6.72 | -8.78 | 19.04 |
| Duration of grip | -3.31 | 5.94 | -16.95 | 7.73 | 3.86 | 5.85 | -7.15 | 17.36 |
| Recovery time | -3.30 | 6.05 | -16.53 | 8.41 | 3.99 | 6.19 | -7.28 | 17.91 |
| Value estimation | -3.38 | 6.03 | -16.58 | 7.55 | 3.79 | 5.91 | -7.32 | 16.77 |

**Supplementary Table 4. Conditional effects of handgrip variables between groups (DS and non-DS).**

*Note:* HGS, Handgrip strength; SE, Standard Error; CI, Confidence Interval.

**Supplementary table 5. The quality of the included studies was assessed with the Newcastle-Ottawa Scale.**

| **Authors, year** | **Selection** | **Comparability** | **Outcome** | **Score** | **Risk of bias** |
| --- | --- | --- | --- | --- | --- |
| **Cross-sectional studies** | | | | | |
| Balic et al (2000) | 2 | 1 | 2 | 5 | High |
| Godoy et al (2005) | 3 | 1 | 2 | 6 | Medium |
| Beqaj et al (2018) | 2 | 1 | 2 | 5 | High |
| Bunsawat et al (2016) | 2 | 1 | 2 | 5 | High |
| Cabeza-Ruiz et al (2009) | 2 | 1 | 2 | 5 | High |
| Cabeza-Ruiz et al (2019) | 2 | 2 | 2 | 6 | Medium |
| Chen et al (2014) | 2 | 1 | 2 | 5 | High |
| Chen et al (2018) | 2 | 1 | 2 | 5 | High |
| Chen et al (2019) | 2 | 1 | 2 | 5 | High |
| Chen et al (2021) | 2 | 1 | 2 | 5 | High |
| Coelho-Junior et al (2019) | 3 | 1 | 2 | 6 | Medium |
| Fedrigo et al (2023) | 2 | 2 | 2 | 6 | Medium |
| Fernhall et al (2003) | 2 | 2 | 2 | 6 | Medium |
| Figueroa et al (2005) | 2 | 2 | 2 | 6 | Medium |
| Heffernan et al (2005) | 2 | 1 | 2 | 5 | High |
| Heffernan et al (2009) | 2 | 1 | 2 | 5 | High |
| Izquierdo-Gomes et al (2013) | 2 | 2 | 2 | 6 | Medium |
| Matute-Llorente et al (2017) | 1 | 1 | 2 | 4 | High |
| Melo et al | 3 | 2 | 2 | 7 | Low |
| Morris et al (1982) | 2 | 2 | 2 | 6 | Medium |
| Priosti et al (2013) | 3 | 1 | 2 | 6 | Medium |
| John et al (2016) | 2 | 1 | 2 | 5 | High |
| Siyah et al (2023) | 2 | 1 | 2 | 5 | High |
| Telejo-Gonzalez et al (2013) | 1 | 1 | 2 | 4 | High |
| Terblanche et al | 2 | 1 | 2 | 5 | High |
| **Cohort studies** | | | | | |
| Pessoa et al (2023) | 3 | 1 | 3 | 7 | Low |
| Pino-Valenzuela et al (2023) | 3 | 1 | 3 | 7 | Low |
| Rosety-Rodrigues et al (2021) | 4 | 1 | 2 | 7 | Low |
| Suares Villadat et al (2019) | 4 | 1 | 3 | 8 | Low |

**Supplementary table 6.** PRISMA Check list.

| **Section and Topic** | **Item #** | **Checklist item** | **Location where item is reported** |
| --- | --- | --- | --- |
| **TITLE** | | |  |
| Title | 1 | Identify the report as a systematic review. | 2 |
| **ABSTRACT** | | |  |
| Abstract | 2 | See the PRISMA 2020 for Abstracts checklist. | 2 |
| **INTRODUCTION** | | |  |
| Rationale | 3 | Describe the rationale for the review in the context of existing knowledge. | 3,4 |
| Objectives | 4 | Provide an explicit statement of the objective(s) or question(s) the review addresses. | 4 |
| **METHODS** | | |  |
| Eligibility criteria | 5 | Specify the inclusion and exclusion criteria for the review and how studies were grouped for the syntheses. | 4,5 |
| Information sources | 6 | Specify all databases, registers, websites, organisations, reference lists and other sources searched or consulted to identify studies. Specify the date when each source was last searched or consulted. | 4 |
| Search strategy | 7 | Present the full search strategies for all databases, registers and websites, including any filters and limits used. | 4 and Supplementary table 1 |
| Selection process | 8 | Specify the methods used to decide whether a study met the inclusion criteria of the review, including how many reviewers screened each record and each report retrieved, whether they worked independently, and if applicable, details of automation tools used in the process. | 4 |
| Data collection process | 9 | Specify the methods used to collect data from reports, including how many reviewers collected data from each report, whether they worked independently, any processes for obtaining or confirming data from study investigators, and if applicable, details of automation tools used in the process. | 5 |
| Data items | 10a | List and define all outcomes for which data were sought. Specify whether all results that were compatible with each outcome domain in each study were sought (e.g. for all measures, time points, analyses), and if not, the methods used to decide which results to collect. | 5 |
|  | 10b | List and define all other variables for which data were sought (e.g. participant and intervention characteristics, funding sources). Describe any assumptions made about any missing or unclear information. | 5,6 |
| Study risk of bias assessment | 11 | Specify the methods used to assess risk of bias in the included studies, including details of the tool(s) used, how many reviewers assessed each study and whether they worked independently, and if applicable, details of automation tools used in the process. | 5 |
| Effect measures | 12 | Specify for each outcome the effect measure(s) (e.g. risk ratio, mean difference) used in the synthesis or presentation of results. | 5,6 |
| Synthesis methods | 13a | Describe the processes used to decide which studies were eligible for each synthesis (e.g. tabulating the study intervention characteristics and comparing against the planned groups for each synthesis (item #5)). | 6 |
|  | 13b | Describe any methods required to prepare the data for presentation or synthesis, such as handling of missing summary statistics, or data conversions. | 5,6 |
|  | 13c | Describe any methods used to tabulate or visually display results of individual studies and syntheses. | 5,6 |
|  | 13d | Describe any methods used to synthesize results and provide a rationale for the choice(s). If meta-analysis was performed, describe the model(s), method(s) to identify the presence and extent of statistical heterogeneity, and software package(s) used. | 6 |
|  | 13e | Describe any methods used to explore possible causes of heterogeneity among study results (e.g. subgroup analysis, meta-regression). | 6 |
|  | 13f | Describe any sensitivity analyses conducted to assess robustness of the synthesized results. | 6 |
| Reporting bias assessment | 14 | Describe any methods used to assess risk of bias due to missing results in a synthesis (arising from reporting biases). | N/A |
| Certainty assessment | 15 | Describe any methods used to assess certainty (or confidence) in the body of evidence for an outcome. | N/A |
| **RESULTS** | | |  |
| Study selection | 16a | Describe the results of the search and selection process, from the number of records identified in the search to the number of studies included in the review, ideally using a flow diagram. | 6 |
|  | 16b | Cite studies that might appear to meet the inclusion criteria, but which were excluded, and explain why they were excluded. | 6 |
| Study characteristics | 17 | Cite each included study and present its characteristics. | 6/7 |
| Risk of bias in studies | 18 | Present assessments of risk of bias for each included study. | 9 |
| Results of individual studies | 19 | For all outcomes, present, for each study: (a) summary statistics for each group (where appropriate) and (b) an effect estimate and its precision (e.g. confidence/credible interval), ideally using structured tables or plots. | 7/8 and Supplementary table 2 |
| Results of syntheses | 20a | For each synthesis, briefly summarise the characteristics and risk of bias among contributing studies. | 9 and Supplementary table 5 |
|  | 20b | Present results of all statistical syntheses conducted. If meta-analysis was done, present for each the summary estimate and its precision (e.g. confidence/credible interval) and measures of statistical heterogeneity. If comparing groups, describe the direction of the effect. | 8 and Supplementary table 2, 3 and 4 |
|  | 20c | Present results of all investigations of possible causes of heterogeneity among study results. | 8 |
|  | 20d | Present results of all sensitivity analyses conducted to assess the robustness of the synthesized results. | 8 and Supplementary table 2, 3 and 4 |
| Reporting biases | 21 | Present assessments of risk of bias due to missing results (arising from reporting biases) for each synthesis assessed. | 7 |
| Certainty of evidence | 22 | Present assessments of certainty (or confidence) in the body of evidence for each outcome assessed. | 8 and Supplementary table 2, 3 and 4 |
| **DISCUSSION** | | |  |
| Discussion | 23a | Provide a general interpretation of the results in the context of other evidence. | 9,10,11 |
|  | 23b | Discuss any limitations of the evidence included in the review. | 11,12 |
|  | 23c | Discuss any limitations of the review processes used. | 12 |
|  | 23d | Discuss implications of the results for practice, policy, and future research. | 11 |
| **OTHER INFORMATION** | | |  |
| Registration and protocol | 24a | Provide registration information for the review, including register name and registration number, or state that the review was not registered. | 12 |
|  | 24b | Indicate where the review protocol can be accessed, or state that a protocol was not prepared. | 12 |
|  | 24c | Describe and explain any amendments to information provided at registration or in the protocol. | 12 |
| Support | 25 | Describe sources of financial or non-financial support for the review, and the role of the funders or sponsors in the review. | 12 |
| Competing interests | 26 | Declare any competing interests of review authors. | 12 |
| Availability of data, code and other materials | 27 | Report which of the following are publicly available and where they can be found: template data collection forms; data extracted from included studies; data used for all analyses; analytic code; any other materials used in the review. | 12 |

*From:*  Page MJ, McKenzie JE, Bossuyt PM, Boutron I, Hoffmann TC, Mulrow CD, et al. The PRISMA 2020 statement: an updated guideline for reporting systematic reviews. BMJ 2021;372:n71. doi: 10.1136/bmj.n71

For more information, visit: <http://www.prisma-statement.org/>
